# Mechanisms and kinetic assays of aminoacyl‐tRNA synthetases

**DOI:** 10.1002/1873-3468.70098

**Published:** 2025-07-04

**Authors:** Igor Zivkovic, Morana Dulic, Ita Gruic‐Sovulj

**Affiliations:** ^1^ Department of Chemistry, Faculty of Science University of Zagreb Zagreb Croatia

**Keywords:** aminoacyl‐tRNA synthetases, AARS editing, AARS kinetic assays, aminoacyl transfer step, amino acid activation, ATP‐PPi exchange, pre‐transfer editing, post‐transfer editing, deacylation assay, aminoacyl‐tRNA synthetase kinetic artefacts

## Abstract

Accurate protein synthesis is crucial for life. The key players are aminoacyl‐tRNA synthetases (AARSs), which read the genetic code by pairing cognate amino acids and tRNAs. AARSs establish high amino acid selectivity by employing physicochemical limits in molecular recognition. However, chemical and structural resemblance between some amino acids prevents their efficient discrimination by AARSs. In these cases, AARSs hydrolyze the formed non‐cognate intermediates or aa‐tRNAs to ensure selectivity, establishing complex reaction pathways within the synthetic and editing sites. Understanding AARS mechanisms is crucial for understanding their biology. Here, we review kinetic assays for exploring AARS mechanisms. For each assay, we state the most suitable substrates, product(s) recommended to follow, and, importantly, possible caveats that may lead to the kinetic artefact.

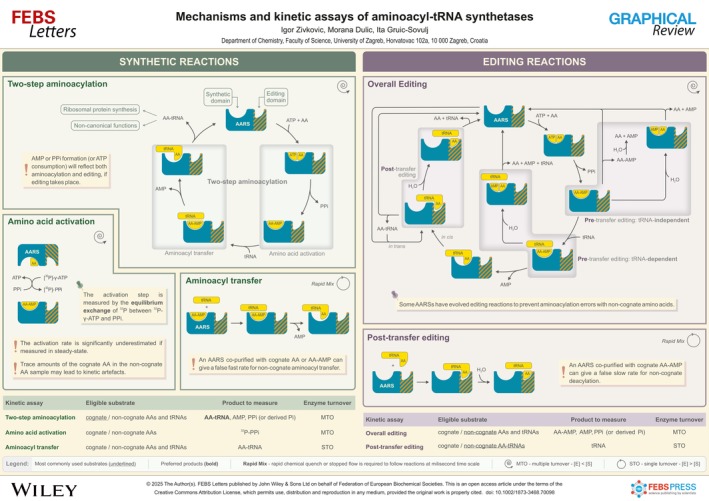

## Graphical abstract

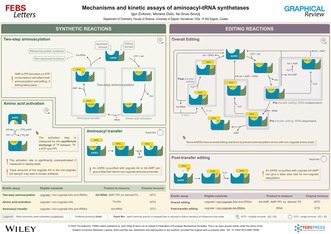

**Abbreviations:**
AA‐AMP: aminoacyl‐adenylate
AARS: aminoacyl‐tRNA synthetase
AMP: adenosine 5’‐monophosphate
ATP: adenosine 5’‐triphosphate
IleRS: isoleucyl‐tRNA synthetase
PPi: pyrophosphate
tRNA: transfer RNA

Aminoacyl‐tRNA synthetases (AARSs) produce aminoacylated tRNAs for protein synthesis [1]. They are divided into two classes [2], each catalyzing the same two‐step reaction, where the amino acid is activated by ATP forming aminoacyl‐adenylate (AA‐AMP) intermediate and pyrophosphate (PPi), followed by the transfer of the aminoacyl moiety to the 3' end of the cognate tRNA and the release of AMP [1]. To maintain accurate translation, some AARSs have evolved editing reactions to prevent aminoacylation errors with non‐cognate amino acids [3]. Pre‐transfer editing occurs in the synthetic site and features dissociation or hydrolysis of non‐cognate AA‐AMP in the absence or presence of tRNA. In contrast, post‐transfer editing comprises the hydrolysis of misacylated tRNA at the dedicated editing domain or by freestanding trans‐acting factors [4]. AARSs present a unique model for studying the evolution of enzyme selectivity and the genetic code. Further, as housekeeping enzymes with evolutionarily distinct features in different domains of life, AARSs are promising targets for antimicrobial inhibitors [5]. Finally, AARS roles in diseases [6] and their non‐canonical roles in cells [7] are rapidly emerging. To link AARS complex catalysis to their biology, one may follow cumulative pathways or independent catalytic steps as described here.

## Two‐step aminoacylation

Aminoacylation is measured in the presence of all substrates, amino acid, tRNA and ATP, following multiple enzyme turnovers under steady‐state conditions. Less frequently used pre‐steady‐state approaches, such as the burst of a product or a catalytic single‐turnover, are valuable for providing a cumulative rate of the steps preceding product dissociation [8, 9], which limits the aminoacylation rate in class I AARSs [10, 11]. Straightforward methods to measure the reaction rate are following the accumulation of AA‐tRNA using a radiolabeled amino acid [12] or tRNA [13] or employing LC‐MS to separate AA‐tRNA from tRNA [14]. If the aminoacylation rate is measured by the formation of PPi, AMP or ATP consumption, it is important to note that these approaches may reflect futile editing cycles (Table 5 in [15]). This is particularly relevant for non‐cognate substrates [15]. The requirement for high‐throughput analyses and the reluctance to use radiolabeled substrates led to various approaches that couple aminoacylation products with another assay(s). The experimental output of these assays is reflected as a change in absorbance associated with changes in either PPi [16, 17] or AMP [18, 19, 20] concentrations [reviewed in 21].

## Amino acid activation

Measuring the activation step is challenging because the intermediate, AA‐AMP, dissociates slowly from the active site (approximately 5×10^‐3^ s^‐1^ [22]). This prevents measurements in steady‐state, where the slow dissociation limits the observed reaction rate. To overcome this, activation is commonly measured via equilibrium exchange, which bypasses the need for product (intermediate) release. The ATP‐PPi exchange assay [23] originally used trace amounts of [^32^P]‐PPi to monitor formation of [^32^P]‐ATP in a reaction mixture containing the enzyme, ATP, unlabeled PPi, and the amino acid. Recently, the assay has been adapted to use more accessible γ‐[^32^P]‐ATP by tracking the production of [^32^P]‐PPi instead. The ATP‐PPi exchange is suitable for cognate and non‐cognate amino acids and the determination of non‐cognate substrates’ discrimination factors. It is important to note that trace amounts of the cognate amino acid in a non‐cognate amino acid sample may lead to an underestimated discrimination [11, 24, 25]. tRNA is not required for the amino acid activation by most AARS, exceptions are described in [27, 28, 29, 30].

Importantly, significant discrepancies are observed between the rates measured by ATP‐PPi exchange and those determined from steady‐state formation of AA‐AMP, AMP or PPi. For example, ATP‐PPi exchange returns the rate of 18 s^‐1^ for isoleucine activation by IleRS. If the reaction is monitored by AMP formation in steady‐state, the rate drops drastically to 0.002 s^‐1^ (Table 1 in [31]). The slow rate reflects enzymatic hydrolysis of cognate AA‐AMP or its dissociation and hydrolysis in solution [32]. Monitoring the activation of a non‐cognate amino acid using this steady‐state approach reflects the rate of non‐cognate AA‐AMP hydrolysis, which is faster than cognate AA‐AMP hydrolysis, but still slower than the chemical step of the non‐cognate amino acid activation [33]. The important message is that while the steady‐state assay, opposite to the [^32^P]‐ATP‐PPi exchange, may offer simpler non‐radioactive monitoring via PPi or AMP detection [16, 17, 18, 19, 20], it reflects slow enzyme regeneration steps and thus significantly underestimates activation rates. However, slow enzyme regeneration does not limit the rate of AA‐AMP formation in the pre‐steady state phase of the reaction [34].

## Aminoacyl transfer

The second step of aminoacylation is measured independently by mixing a pre‐formed [35] or *in situ*‐formed [33] AARS:AA‐AMP complex with limiting amounts of tRNA, thereby restricting catalysis to a single turnover. The half‐time of the reaction usually falls within the millisecond range, requiring rapid mixing techniques such as rapid chemical quench or stopped flow. The rate is obtained following AA‐tRNA formation, using radiolabeled tRNA (preferred) or amino acid. The transfer step can be tested with cognate and non‐cognate substrates. An enzyme with an inactivated editing domain is required for non‐cognate amino acids. Enzyme purity is important due to the typically used high enzyme concentration: an AARS co‐purified with amino acid or AA‐AMP can lead to kinetic artefacts [11].

## Editing reactions

The editing assay (ATPase assay) measures overall ATP turnover by monitoring ATP consumption or the formation of AMP or PPi in the presence of AARS and tRNA [11]. To specifically measure tRNA‐independent editing, tRNA must be omitted from the reaction. Measuring tRNA‐dependent pre‐transfer editing requires tRNA and the AARS with an inactivated editing domain. However, under these conditions, AMP formation originates from both pre‐transfer editing and misacylation by the post‐transfer editing‐deficient AARS. To compare these contributions, editing (AMP production) and misacylation (AA‐tRNA production) can be tested in parallel reactions [36, 37]. The ratio of rate constants higher than 1 in favor of AMP indicates the existence of tRNA‐dependent pre‐transfer editing. Editing can also be tested with cognate amino acids, but these reactions are slow and negligible for wild‐type AARSs [38].

## Post‐transfer editing

Post‐transfer editing (deacylation) is followed by mixing AARS with a misacylated tRNA pre‐formed by using either a labeled amino acid or tRNA (preferred) [37]. AARS used for preparative misacylation should be free of any co‐purified cognate amino acid or AA‐AMP [11]. Single‐turnover conditions (AARS in excess of AA‐tRNA) are recommended to avoid rate‐limiting product dissociation. Deacylation rates are usually fast and obtained following tRNA product formation [37] or AA‐tRNA substrate consumption [39] using rapid mixing techniques. Caution is advised regarding the enzyme purity, as cognate AA‐AMP co‐purified with the enzyme can contribute to aminoacylation of the deacylated tRNA, thereby masking deacylation activity [11].

## Author contributions:

IZ made a poster part, MD drafted a text part, IGS designed and edited the review
